# Physical therapy aimed at self-management versus usual care physical therapy after hip arthroscopy for femoroacetabular impingement: study protocol for a randomized controlled trial

**DOI:** 10.1186/s13063-016-1222-7

**Published:** 2016-02-17

**Authors:** M. Tijssen, R. E. H. van Cingel, J. B. Staal, S. Teerenstra, E. de Visser, M. W. G. Nijhuis-van der Sanden

**Affiliations:** Sports Medical Center Papendal, Scientific Institute for Health Sciences, IQ Healthcare, Papendallaan 7, 6816 VD Arnhem, The Netherlands; Radboud University Medical Center, Scientific Institute for Health Sciences, IQ Healthcare, PO Box 9101, 114 IQ, 6500 HB Nijmegen, The Netherlands; HAN University of Applied Sciences, Research Group Musculoskeletal Rehabiliation, PO Box 6960, 6503 GL, Nijmegen, The Netherlands; Department for Health Evidence, Radboud Institute for Health Sciences, Section Biostatistics, Radboud University Medical Center, PO Box 9101, 133 HEV, 6500 HB Nijmegen, The Netherlands; Rijnstate Hospital Arnhem, Wagnerlaan 55, 6815 AD Arnhem, The Netherlands; Scientific Institute for Health Sciences, IQ Healthcare, Radboud University Medical Center, PO Box 9101, 114 IQ, 6500 HB Nijmegen, The Netherlands

**Keywords:** Hip joint, Femoroacetabular impingement, Arthroscopy, Rehabilitation, Physical therapy

## Abstract

**Background:**

Femoroacetabular impingement has been recognized as a common cause of hip pain and dysfunction, especially in athletes. Femoroacetabular impingement can now be better treated by hip arthroscopy but it is unclear what postoperative rehabilitation of hip arthroscopy should look like. Several rehabilitation protocols have been described, but none presented clinical outcome data. These protocols also differ in frequency, duration and level of supervision. We developed a rehabilitation protocol with supervised physical therapy which showed good clinical results and is considered usual care in our treatment center. However, it is unknown whether, due to the relatively young age and low complication rate of hip arthroscopy patients, rehabilitation based on self-management might lead to similar results. The aims of this pilot study are (1) to determine feasibility and acceptability of the self-management intervention, (2) to obtain a preliminary estimate of the difference in effect between physical therapy aimed at self-management versus usual care physical therapy in patients who undergo hip arthroscopy for femoroacetabular impingement.

**Methods/Design:**

Thirty participants (aged 18–50 years) scheduled for hip arthroscopy will be included and randomized (after surgery) to either self-management or usual care physical therapy in this assessor-blinded randomized controlled trial. After surgery, the self-management group will perform a home-based exercise program three times a week and will receive physical therapy treatment once every 2 weeks for 14 weeks. The usual care group will receive physical therapy treatment twice a week for 14 weeks and will perform an additional home-based exercise program once a week. Assessment will occur preoperatively and at 6, 14, 26 and 52 weeks after surgery. Primary outcomes are feasibility, acceptability and preliminary effectiveness. Feasibility and acceptability will be determined by the willingness to enroll, recruitment rate, adherence to treatment, patient satisfaction, drop-out rate and adverse events. Preliminary effectiveness will be determined using the following outcomes: the International Hip Outcome Tool 33 and hip functional performance as measured with the Single Leg Squat Test 14 weeks after surgery.

**Discussion:**

The results of this study will be used to help decide on the need, feasibility and acceptability of a large-scale randomized controlled trial.

**Trial registration:**

This protocol was registered with the Dutch Trial Registry (NTR5168) on 8 May 2015.

**Electronic supplementary material:**

The online version of this article (doi:10.1186/s13063-016-1222-7) contains supplementary material, which is available to authorized users.

## Background

Intra-articular hip pathology has gained increasing interest over the past decade [1]. In particular, femoroacetabular impingement (FAI) has been recognized as common cause of hip pain and dysfunction [1, 2]. The incidence of FAI in the general population has been reported to range from 4 % in healthy women to 24 % in healthy men [3, 4]. Moreover, 23 % of people with radiographically confirmed FAI complain of hip pain [5]. FAI occurs when the proximal femoral head does not permit normal range of motion in the acetabular socket [2]. This impingement can be based on abnormal morphology of the femoral head (cam impingement), acetabular rim (pincer impingement) or both [2]. FAI can cause other intra-articular hip pathology, such as labral pathology and chondral damage [2]. It is also thought to lead to development of secondary osteoarthritis of the hip [3, 6–9]. One of the most commonly used options to treat FAI over recent years has been hip arthroscopy [1]. This arthroscopic technique is often performed to treat intra-articular hip pathology and the number of procedures performed has increased considerably over the last 10 years [1]. Due to the development of hip arthroscopy, FAI can now be better treated with fewer complications and a faster rehabilitation rate [10, 11].

It is unclear which type of rehabilitation is most beneficial for the postoperative FAI population. Several postoperative rehabilitation protocols have been described which all include physical therapy treatment and exercises [10, 12–19]. Yet, therapy goals, frequency and duration of these protocols differ [10, 12–19]. More importantly, the studies describing these rehabilitation protocols provide little to no information with regard to clinical outcome data [11]. Only a few case studies have described clinical outcome data for postoperative interventions in hip arthroscopy patients [10, 14, 16, 17]. So, the clinician can choose from different rehabilitation protocols, but there is little information on the effects achieved. Based on the differences in existing rehabilitation protocols and the lack of clinical outcome data we developed a rehabilitation protocol for hip arthroscopy patients. This protocol combines information retrieved from the available medical literature on postoperative rehabilitation with the clinical experience of the lead researcher (MT) and orthopedic surgeon (EV) [10–19]. The protocol has been satisfactorily used as usual care in clinical practice over the last 5 years [20]. Current results of this protocol show that at a mean follow-up time of 2.3 years after surgery, 81 % of patients reported improvement on the Global Perceived Effect (GPE) Scale and 84 % returned to sports activities. A full recovery of hip functional performance, as measured with balance and hop tests, was established [20].

The majority of the available rehabilitation protocols (including our own) are based on supervised physical therapy with a small, additional home-based exercise program. A self-management strategy (i.e., increasing the home-based exercise program and decreasing supervision) would lead to a more cost-effective and widely applicable rehabilitation [11, 20]. Rehabilitation based on self-management might be adequate as hip arthroscopy is often performed in a young to middle aged, healthy population with little risk of complications. Until now this has not been prospectively investigated. Currently, one randomized controlled trial into the efficacy of postoperative physical therapy for FAI is underway [21]. However, these authors compare physical therapy versus a control group (one in-hospital physical therapy visit combined with an information brochure) instead of a self-management group [21]. A comparison between physical therapy aimed at self-management and usual care physical therapy in patients treated for FAI by means of hip arthroscopy seems warranted. Because of the lack of earlier randomized controlled trials (RCTs) in this field executing a pilot controlled study into the feasibility, acceptability and preliminary effectiveness is necessary before planning and conducting a larger-scale RCT [22].

The aims of this pilot study are (1) to determine feasibility and acceptability of the self-management intervention, (2) to obtain a preliminary estimate of the difference in effect between two rehabilitation strategies, self-management versus usual care physical therapy (according to the developed protocol), in patients who undergo hip arthroscopy for FAI.

## Methods/Design

### Study design

This study protocol describes a parallel-designed, two-arm, assessor-blinded RCT. Outcomes will be assessed at 6, 14, 26 and 52 weeks after surgery in which the 14-week assessment will be the main outcome assessment. The study protocol has been developed based on the Standard Protocol Items: Recommendations for Interventional Trials (SPIRIT) guidelines (see Additional file 1) [23]. The study design was approved by the local ethics committee; Commissie Mensgebonden Onderzoek (CMO) Arnhem-Nijmegen (2015-1730) and registered with the Dutch Trial Registry (NTR5168) on 8 May 2015. All participants will be asked to sign informed consent before start of the study (see Additional file 2).

### Participants

A total of 30 participants (18–50 years of age) scheduled for hip arthroscopy at Rijnstate Hospital, Arnhem, The Netherlands, and living in the near proximity of this hospital (less than 50 kilometers) will be included in this study. Participants are eligible if (1) they have experienced hip/groin pain for at least 3 months, (2) are diagnosed with FAI by one of two orthopedic surgeons (ET/MW) based on symptoms, clinical signs and imaging findings [24], (3) are willing to sign informed consent, and (4) are willing to participate in the rehabilitation program at Sports Medical Center Papendal (SMCP), Arnhem, The Netherlands. Participants will be excluded if (1) they are professional athletes, (2) there is radiographic evidence of hip osteoarthritis (more than Tonnis grade 1:3), (3) there are contra-indications for the hip arthroscopy procedure, (4) there are other pathologies, such as cardiovascular disease, that can influence therapy effects, (5) there is an inability to speak or understand the Dutch language, and (6) there is an inability to comply with postoperative rehabilitation and exercises due to other reasons, such as a lack of time.

### Study procedure

**Fig. 1:**
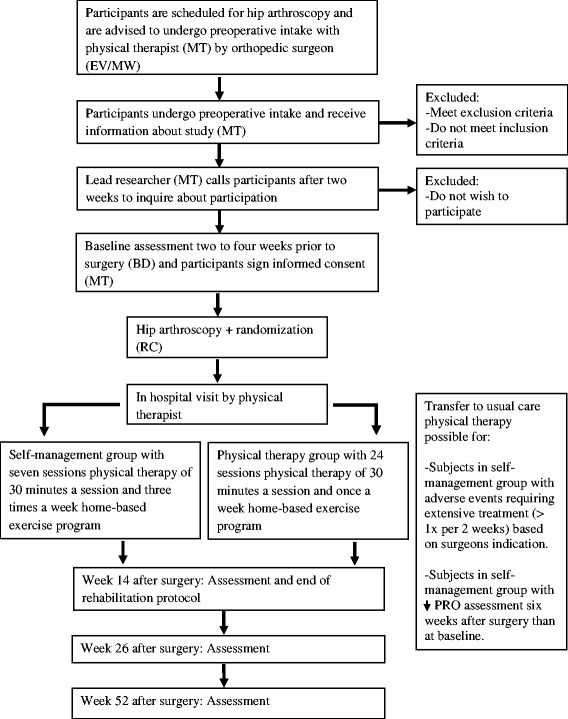
Flow chart of study procedure

Potential participants will be identified by the orthopedic surgeons (EV/MW) and will be advised to undergo a preoperative intake assessment with a physical therapist (MT) at SMCP, Arnhem, The Netherlands. This is part of usual preoperative care. At the preoperative intake assessment all participants will be informed about the study (including information on both interventions). Two weeks after this assessment participants will be contacted by the lead researcher (MT) in order to inform the researcher whether they want to participate in the study. If so, they are invited for a baseline assessment 2 to 4 weeks before surgery. At this assessment (BD) they will also receive instructions about direct postoperative treatment and sign an informed consent (MT). Surgery will be performed by one of two surgeons (EV/MW) at Rijnstate Hospital, Arnhem, The Netherlands. Randomization will occur directly after surgery. Participants will be divided into two groups (self-management group versus usual care physical therapy group) which will both be treated by the same physical therapist (MT). The self-management group will receive physical therapy treatment once every 2 weeks (weeks 2, 4, 6, 8, 10, 12 and 14) leading to a total of seven sessions in 14 weeks whereas the usual care physical therapy group will receive physical therapy treatment twice a week over 14 weeks (24 sessions). The self-management group will be asked to perform an additional home-based exercise program three times per week. The usual care physical therapy group will be asked to perform a similar program once a week. Participants in the self-management group who report a deterioration on the International Hip Outcome Tool 33 (IHOT-33) at 6 weeks after surgery compared to the baseline/preoperative measurement or who experience complications from surgery, as described in Fig. 1, will be offered a transition to the usual care physical therapy group. In case of (serious) adverse events further participation of the study will be decided on by consultation with the responsible surgeon and participant. No adverse events or serious adverse events are expected. In case of (serious) adverse events the responsible surgeon will be in charge of treatment immediately. All adverse events will be documented by the main researcher (MT). Re-assessment will be performed by one blinded assessor (BD) and will occur at 6, 14, 26 and 52 weeks after surgery. A flow chart of the study procedure is shown in Fig. 1.

### Blinding and randomization

The surgeons (EV/MW) and assessor (BD) executing the assessments will be blinded to group allocation. The statistician (ST) will be blinded to group allocation until completion of the statistical analysis. However, it is impossible to blind the physical therapist (MT) executing the rehabilitation protocol and the study participants. Participants will be asked not to reveal group allocation when visiting the orthopedic surgeon postoperatively as well as when undergoing follow-up measurements by the blinded assessor (BD). Before randomization, participants will be asked to state group preference. This information will be used to later investigate whether group preference influenced study results.

Randomization is done on the individual level through a computer-generated random-sequence table. Pre-stratification is applied for gender. Opaque, sequentially numbered, sealed envelopes are prepared for each stratum (that is, gender) by a researcher (RC) who is not involved in enrolling the participants, in assigning them to their groups or performing follow-up measurements. Every envelope will contain a paper indicating the treatment allocation. Participants will receive their envelope during the first consultation with the physical therapist after surgery (2 weeks postoperative).

### Hip arthroscopy procedure and immediate postoperative care

Arthroscopy will be performed by one of two orthopedic surgeons (EV/MW) with respectively 10 and 3 years of experience in this field of expertise. Spinal needles are placed under image intensifier control to mark the anterior and anterolateral portals. Guide wires and cannulated trocars will be used to introduce cannulae, arthroscopes, and other instruments. A 70° arthroscope will be used to adequately visualize the acetabulum, acetabular labrum, ligaments and the anterior, superior, and posterior aspects of the femoral head. These areas of the hip will be inspected and also probed to assess labral attachment and articular cartilage softening. Pincer-type impingement is typically found in the superior anterior quadrant and will be identified when there is bone overgrowth, a pincer projection causing labral displacement or a crossing sign to be seen over the labrum with fluoroscopy. In order to establish cam-type impingement, traction will be released and the peripheral compartment will be investigated. Cam-type impingement will be defined during arthroscopic physical examination, especially during flexion and internal rotation and by the presence of local abnormalities coherent with cam-type impingement, such as chondral lesions. In all cases in which surgically treatable pathology is identified such treatment will be performed arthroscopically. Immediate postoperative care will be the same for both groups. Participants will stay in the hospital for one night. They will receive a visit from the physical therapist in the hospital in order to improve gait function with crutches and obtain initial advice for the first postoperative week at home. A follow-up visit with the orthopedic surgeon will be scheduled 6 weeks after surgery.

### Study interventions

Physical therapy treatment at SMCP will start 2 weeks after surgery for both groups. For the first two postoperative weeks both groups will start self-mobilizations and basic stability exercises unsupervised on a daily basis at home as explained to them preoperatively and during immediate postoperative care in the hospital.

#### Self-management group

The self-management group will conduct exercises three times a week at home with supervision and treatment by a physical therapist once every 2 weeks. The content of the therapy will be exactly the same as for the usual care physical therapy group, except for the frequency of the meetings between the participant and the physical therapist. This means that the amount of hands-on physical therapy, as well as instructions concerning adjustments to the exercises and education, will differ.

#### Usual care physical therapy group

The usual care physical therapy group will receive hands-on physical therapy care and conduct exercises supervised by a therapist twice a week and unsupervised (at home) once a week.

#### Content of postoperative rehabilitation protocol

The content of the physical therapy protocol consists of hands-on physical therapy care, exercises, education, cardiovascular training and return to sports. This protocol is based on previous medical literature combined with our own clinical experience [10–20]. For a complete overview of the postoperative rehabilitation protocol for both groups see Tables 1, 2 and 3 and Additional file 3A/B [20]. The exact content of each therapy session will be reported in the therapy records. Treatment that is delivered, but also treatment that has not been delivered (including reasons why), will be reported at every session by the physical therapist.

**Table 1:** Overview of postoperative rehabilitation protocol – hands-on physical therapy care [25]

Technique	Aim	Description	Timeframe	Dosage
Soft tissue massage and trigger point therapy of iliopsoas, rectus femoris, sartorius, adductor group, gluteus medius/minimus, tensor fascia latae and quadratus lumborum muscles	Address soft tissue restrictions with the aim of pain reduction and mobility improvement of the hip and pelvis	Sustained pressure to each trigger point (with muscle on stretch). Longitudinal massage along the muscle belly	Week 2 - 14	30–60 seconds per trigger point < 5 minutes per muscle
Manual mobilizations of the hip	To improve mobility and pain-free movement of the hip (especially flexion and internal/external rotation)	Traction directed inferior with hip in maximum loose packed position. Traction applied with traction belt directed inferiorly/laterally with hip in flexion (and, if necessary, rotations)	Week 2 – 8	3–5 sets 30–60 seconds
Manual mobilizations of the lumbar spine	To improve mobility and pain-free movement of the hip and lumbar spine	Unilateral posterior-anterior accessory glides grade 3 or 4. Gentle mobilizations with subject/participant lying on their side	Week 2 – 8	3–5 sets 30–60 seconds
Manual mobilizations of the pelvis	To improve mobility and pain-free movement of the hip and pelvis	Mobilizations of the ilium in the anterior or posterior direction or mobilization of the sacrum	Week 2 – 8	3–5 sets 30–60 seconds

The physical therapy protocol is performed by one physical therapist (MT) and is semi-structured. The hands-on physical therapy care will be based on subject specific indications and clinical presentation such as pain and range of motion (ROM) restrictions. In case multiple techniques are indicated the order will be as follows: manual mobilizations of lumbar spine, pelvis and hip before soft tissue massage and trigger point therapy

**Table 2:** Overview of postoperative rehabilitation protocol – exercises [20, 21, 25–28]

Exercise	Aim	Description	Timeframe	Dosage
Self-mobilizations of the hip, pelvis and lumbar spine	To help improve mobility and pain-free movement of the hip, pelvis and lumbar spine and prevent adhesions of the hip capsule	See Additional file 3A; row 1 exercises 1–5 See Additional file 3A; row 1 exercise 6	Weeks 0–2 Weeks 2–8	1 minute per exercise, 3 times per day 1 minute per exercise
Anterior and posterior hip stretch	To help improve hip flexion and extension mobility	See Additional file 3A; row 2 exercises 1–2	Weeks 28	3–5 sets 30 seconds
Hip muscle retraining	To optimize neuromuscular control and stability of the hip	See Additional file 3A; row 3 exercises 1–5	Weeks 0–4	3 sets 12–20 repetitions
Hip muscle strengthening (focus on extensor/rotator strengthening)	To optimize neuromuscular control, stability and strength of the hip	See Additional file 3A; row 4–5 exercises 1–9	Weeks 4–14	3 sets 8–12 repetitions with increasing load based on experienced fatigue
Functional hip muscle strengthening	To optimize neuromuscular control, stability and strength of the hip in patient specific (sport) activities	Exercises based on patient-specific goals or (sport) demands such as kicking in soccer or throwing/smashing in volleyball/tennis	Weeks 10–14	3 sets 8–12 repetitions with increasing load based on experienced fatigue

The physical therapy protocol is performed by one physical therapist (MT) and is semi-structured. Loads will be adjusted based on the participants functional performance and rehabilitation goals

**Table 3:** Overview of postoperative rehabilitation protocol – cardiovascular training and return to sports [11]

Exercise	Aim	Description	Timeframe	Frequency
Stationary cycling	Improve cardiovascular fitness and hip range of motion	Upright home trainer with set height to avoid hip flexion over 90° (start with 15 minutes) If cycling is main sport or participant does not desire return to (any) sport activities	Week 0 – 4 Week 4 – 14	Daily 3 times a week
Cross trainer	Improve cardiovascular fitness and hip functional performance	Start with 15 minutes at moderate intensity (60–80 % maximum heart rate)	Week 5 – 10	3 times a week
Treadmill/jogging	Improve cardiovascular fitness and hip functional performance	Start with interval training at moderate intensity preferable outside on grass/track	Week 10 – 14	3 times a week
Acceleration/cutting/agility skills	Initiate return to sports performance	Zig-zag jogging, speedladder skills	Week 8 – 12	2 times a week
Sport-specific drills	Initiate return to sports performance	Exercises based on patient specific goals or (sport) demands such as kicking in soccer or throwing/smashing in volleyball/tennis	Week 10 – 14	2 times a week

The physical therapy protocol is performed by one physical therapist (MT) and is semi-structured. Specific return to sport exercises will be tailored for each individual participant based on (1) sport activity (2) desired level of sport activity and (3) current level of function

#### Hands-on physical therapy care

Hands-on physical therapy care consists of manual mobilizations, massage and trigger point therapy by a physical therapist (MT) (Table 1) [25]. These modalities will be performed by the physical therapist based on subject specific indications and clinical presentation such as pain and range of motion (ROM) restrictions. Mobility restrictions and mobility progression will be measured with a goniometer (Fysiosupplies: 20 cm) and reported in the therapy records.

#### Exercises

The exercises consist of strength and stability exercises as well as self-mobilizations of the hip, pelvis and lumbar spine [20, 21, 25–28]. These exercises will be performed statically and dynamically and will be tailored to the participant’s level of fitness. Loads will be adjusted based on the participant’s functional performance and rehabilitation goals. From week 10 these exercises will be adjusted to the specific sports/activity demands of each participant, for example kicking and cutting/pivoting in soccer players. For an overview of exercise progression and exercises see Table 2 and Additional file 3A/B.

#### Education

Education will consist of information on joint protection, postoperative weight-bearing (use of two crutches for 4 weeks starting with flat foot weight-bearing and gradually increasing to full weight-bearing) and regaining complete function in activities of daily life, work and sports as well as information on the importance of the home-based program [11]. The education will start preoperatively (participants will also receive an information booklet prior to surgery) and will continue throughout the complete postoperative rehabilitation. It will be tailored based on the participant’s level of function and knowledge.

#### Cardiovascular training and return to sports

Cardiovascular training will be started by means of a bicycle ergometer for the first 4 weeks in all participants. Participants in the home program, who do not have access to a bicycle ergometer, are offered use of a bicycle ergometer at SMCP, Arnhem, The Netherlands. After 4 weeks a distinction will be made for participants for whom cycling is the main sport or who do not perform sports; they will continue cardiovascular training by means of the bicycle ergometer. All other participants will progress by means of a cross trainer and further the rehabilitation process towards jogging. Specific return to sport exercises will be tailored for each individual participant based on (1) sport activity, (2) desired level of sport activity, and (3) current level of function (Table 3) [11].

### Outcome assessment

The complete rehabilitation will take 14 weeks, excluding the preoperative intake and follow-up assessments. These assessments are all conducted by the same researcher (BD) blinded to group allocation and are conducted at the following time points:

- T0 – preoperative
- T1 – 6 weeks postoperative
- T2 – 14 weeks postoperative
- T3 – 6 months postoperative (26 weeks)
- T4 – 1 year postoperative (52 weeks)

For an overview of outcomes, outcome measures and assessment time points see Table 4.

**Table 4:** Overview of outcomes, outcome measures and assessment time points

Outcomes	Outcome measures	Assessment time point^a^
Feasibility and acceptability		
Number of therapy sessions + exact content of therapy	Therapy records	14, 26, 52 weeks
Adherence home-based exercise program	Log book	14 weeks
Adherence to log book completion	Log book	14 weeks
Willingness to enroll	Study records1 June 2016 (final inclusion date)	
Patient satisfaction	Questionnaire	14 weeks
Eligible patients	Study records1 June 2016 (final inclusion date)	
Recruitment rate	Study records1 June 2016 (final inclusion date)	
Drop-out rate	Questionnaire	14, 24, 52 weeks
Adverse events	Questionnaire	14, 24, 52 weeks
Other treatment/co-interventions	Log book/Questionnaire	14, 26, 52 weeks
Preliminary estimate of effect		
Perceived hip function and health-related QoL^b^	International Hip Outcome Tool 33 (IHOT-33)	0, 6, 14, 26, 52 weeks
Hip functional performance	Single Leg Squat Test (SLST)	0, 6, 14, 26, 52 weeks
Other outcomes		
Activity level	Modified Tegner Activity Scale	0, 14, 26, 52 weeks
Sports activity level	Hip Sports Activity Score (HSAS)	0, 14, 26, 52 weeks
Rating of change	Global Perceived Effect Scale (GPE)	14, 26, 52 weeks
Range of motion	Goniometer	0, 14, 26, 52 weeks
Strength	Hand Held Dynamometer	0, 14, 26, 52 weeks
Hip functional performance hop/jump	Single Leg Hop Test/Star Excursion Balance Test	0, 14, 26, 52 weeks
Patient history	Questionnaire	0 weeks
Patient demographics	Questionnaire	0 weeks
Surgical procedure + exact perioperative diagnosis	Surgical report	Following surgery
Medication use	Questionnaire	0, 14, 26, 52 weeks

^a^Assessment time point = point at which assessment is performed in weeks after surgery or calendar date (in case of study records being the outcome measurement). 0 weeks = preoperative baseline assessment. ^b^ *QoL* quality of life

#### Feasibility and acceptability

Feasibility of the study intervention will be assessed by adherence to the physical therapy program [22]. In order to establish adherence to the physical therapy program the number of therapy sessions will be recorded. Also, the exact content of both therapy interventions (based on therapy records) will be compared. Participants will be asked to fill out a log book in which adherence to the home-based exercise program will be reported as well as exercise intensity, fatigue and experienced pain. This log book will also be used to monitor and account for additional training/sports activities undertaken during the duration of the trial. Both the content of the log book as well as adherence to log book completion will be registered. Acceptability of the study intervention will be assessed evaluating willingness to enroll and by means of a patient satisfaction questionnaire to be answered 14 weeks after surgery [22]. In order to assess feasibility of the study design, the number of eligible patients, recruitment rate, drop-out rate and adverse events will be assessed [22]. Drop-outs and adverse events will be asked for in general questionnaires to be filled out at every assessment. Participants will be asked not to use or undergo treatments other than the ones suggested in this trial or start additional training/sports activities for the duration of the trial. This will be monitored by means of the aforementioned questionnaire as well as the log book.

#### Preliminary estimate of effect

The preliminary estimate of the difference in effect will be determined on health-related quality of life measured by the International Hip Outcome Tool 33 (IHOT-33) and functional performance measured by the Single Leg Squat Test (SLST). The IHOT-33 score consists of 33 questions, regarding hip disease and quality of life, each scored on a Visual Analogue Scale (VAS) with 0 representing the worst and 100 representing the best score [29]. A final score is calculated by summing up the scores of all questions answered and dividing it by the number of questions answered [29]. Earlier studies have shown that this a reliable and valid questionnaire specifically developed for use in a young population with intra-articular hip pathology [30]. The SLST consists of a squat task in which a subject stands on one leg on a 20-cm box with arms folded across their chest. The subject then squats down to a 60° knee angle five times at rate of one squat per 2 seconds [31]. This performance is scored based on five criteria [32] and has shown good inter- and intra-rater reliability in a population of subjects with hip pain [31, 32].

#### Other outcomes

Other outcomes consist of patient-reported outcome (PRO) questionnaires, functional performance tests and general patient information. Three PRO questionnaires will be used, namely the Modified Tegner Activity Scale, the Hip Sports Activity Scale (HSAS) and Global Perceived Effect Scale (GPE). The Modified Tegner Activity Scale measures general physical activity level based on a 0 to 10 scale [33]. The HSAS measures sports activity level on a similar 0 to 10 scale and is specifically developed for hip patients [34]. Both questionnaires have been shown to have good reliability and validity in populations with lower extremity injuries [30]. The GPE will be used to measure the participants’ perceived change. This scale measures perceived change following treatment on a six-point ordinal scale. It has shown good validity in monitoring individual improvement after interventions [35].

In order to establish functional performance the following quantitative measurements will be executed: hip ROM measurements, hip strength measurements, the Single Leg Hop Test and the Star Excursion Balance Test. Range of motion of hip flexion, extension, abduction, adduction, external and internal rotation will be determined with a goniometer (Fysiosupplies: 20 cm) [11, 36]. Strength tests of these same directions are performed with a Hand Held Dynamometer (microFET 2, Hoggan Health Industries, West Jordan, UT, USA) using the make’s method and average outcome of three trials as final score [11, 36]. The Single Leg Hop Test and Star Excursion Balance Test will be executed as described in earlier studies [31]. Both these tests have shown reliability and validity for use in a population of subjects with hip pathology based on recent systematic reviews [31].

General patient information such as patient history, patient demographics, surgical procedure, exact perioperative diagnosis and medication use will be gathered based on questionnaires and surgical reports.

### Data and statistical analysis

Statistical analysis will be performed with IBM SPSS Statistics 22.0. The primary aims are to establish feasibility, acceptability and to obtain an estimate of the difference in effect between the self-management and usual care physical therapy group. A testing strategy for difference in effect of the primary outcomes IHOT-33 and SLST will be pre-specified as follows: first, IHOT-33 will be tested at the 0.05 level and if statistically significant (and only then) SLST will be tested (hierarchical testing at significance level 0.05). The pre-specification allows for valid inference on the primary endpoints. The other endpoints will be analyzed descriptively. An explorative analysis for the effects adjusted for age and subgroup of FAI (as diagnosed perioperatively) will be executed. Changes from baseline to different time points will be analyzed with analysis of covariation (ANCOVA) (baseline as covariate) providing an estimate of the effects and its 95 %-confidence interval. Descriptive statistics including means and standard deviations (SDs) at each time point of each outcome will be reported. Longitudinal analysis using linear mixed models will also be performed.

### Sample size

In line with the aim of obtaining an estimate of the difference in effect between the self-management and physical therapy group, the target sample size aims to achieve a reasonable precision (i.e., half-width of the 95 %-confidence Interval) of this difference at week 14 in the IHOT-33 score using an ANCOVA analysis with baseline value of the outcome measure, IHOT-33, as covariate. We assume a SD of 25 and test-retest reliability of 0.85 [29, 30]. With 15 subjects per group (i.e., 30 subjects in total) this leads to a precision difference of 9.4.

## Discussion

This study provides a protocol for a pilot randomized controlled study into the feasibility, acceptability and preliminary effectiveness of two physical therapy rehabilitation strategies, self-management versus usual care physical therapy, in patients who undergo hip arthroscopy for FAI. This study will identify feasibility and acceptability by means of willingness to enroll, the number of eligible patients, recruitment rate, adherence to treatment, patient satisfaction, possible drop-out rates and adverse events [22]. Additionally it will obtain a preliminary estimate of the difference in effect of the two physical therapy rehabilitation strategies in order to assist in future power calculations for a larger RCT [22].

There is little published clinical evidence to support or refute the use of postoperative rehabilitation in hip arthroscopy patients [8, 11, 20, 21]. The rehabilitation protocol as described in this study is based on information retrieved from the available medical literature on postoperative rehabilitation combined with the clinical experience of the lead researcher (MT) and an orthopedic surgeon (EV) [10–20]. To the authors’ knowledge no studies have been performed into self-management after hip arthroscopy for FAI.

The study was designed based on the principles of a RCT with precision analysis whereby one can expect to find a precision difference between both groups of 9.4. This precision analysis is performed in order to establish data for a larger RCT. The initial outcomes (IHOT-33 and SLST) used to determine a preliminary estimate of the difference in effect are reliable and valid for use in a population of hip arthroscopy patients and are translated and validated into the Dutch language [29–31, Tak et al., 2015 unpublished data]. These outcomes are widely recommended for use in this particular population and will provide data for comparison with other studies such as the aforementioned trial by Bennell et al. [21, 29–31].

The findings of this study will help decide on the need, feasibility and acceptability of the development of a larger RCT for physical therapy in hip arthroscopy patients treated for FAI. Also, the pilot data will give an idea about the effect of postoperative care for hip arthroscopy patients and will possibly help guide clinical decision-making.

## Trial status

This trial is ongoing since the 1 June 2015. At the time of submission of this protocol six subjects have been included in the study over a 6-month recruitment period. To date, none of the participants have completed the follow-up period and no adverse events have been reported.

## Additional files

Additional file 1:
**Standard Protocol Items: Recommendations for Interventional Trials (SPIRIT) checklist.** (DOC 121 kb) Additional file 2:
**Patient Informed Consent (Dutch).** (DOCX 12 kb) Additional file 3:
**(A) Specific examples of exercises included in exercise program.** (B) Example of exercise progression in exercise program. (DOCX 730 kb)

## Abbreviations

CMO: Commissie Mensgebonden Onderzoek
FAI: femoroacetabular impingement
GPE: Global Perceived Effect Scale
HSAS: Hip Sports Activity Scale
IHOT-33: International Hip Outcome Tool
PRO: patient-reported outcome
RCT: randomized controlled trial
ROM: range of motion
SLST: Single Leg Squat Test
SMCP: Sports Medical Center Papendal
SPIRIT: Standard Protocol Items: Recommendations for Interventional Trials
VAS: Visual Analogue Scale
