# Supplementary material for: Physical therapy aimed at self-management versus usual care physical therapy after hip arthroscopy for femoroacetabular impingement: study protocol for a randomized controlled trial
Source: Trials. 2016 Feb 17;17:91. doi: 10.1186/s13063-016-1222-7 (PMC4756499; doi:10.1186/s13063-016-1222-7)
Supplement: Additional file 2: — Patient Informed Consent (Dutch). (DOCX 12 kb) [file 13063_2016_1222_MOESM2_ESM.docx]

**Toestemmingsformulier**

**Revalidatie na een kijkoperatie voor Femoro Acetabulair Impingement (FAI): vergelijking van twee behandelmethoden in een pilotstudie.**

Ik heb de informatiebrief voor de proefpersoon gelezen. Ik kon aanvullende vragen stellen. Mijn vragen zijn genoeg beantwoord. Ik had genoeg tijd om te beslissen of ik meedoe.

Ik weet dat meedoen helemaal vrijwillig is. Ik weet dat ik op ieder moment kan beslissen om toch niet mee te doen. Daarvoor hoef ik geen reden te geven.

Ik geef toestemming om de specialist(en) die mij behandelt te vertellen dat ik meedoe aan dit onderzoek.

Ik weet dat sommige mensen mijn gegevens kunnen zien. Die mensen staan vermeld in de informatiebrief als zijnde hoofdonderzoeker.

Ik geef toestemming om mijn gegevens te gebruiken, voor de doelen die in de informatiebrief staan.

Ik geef wel/geen* toestemming om gegevens nog maximaal 15 jaar na afloop van dit onderzoek te bewaren.

Ik vind het goed om aan dit onderzoek mee te doen.

Naam proefpersoon:

Handtekening: Datum : __ / __ / __

-----------------------------------------------------------------------------------------------------------------

Ik verklaar hierbij dat ik deze proefpersoon volledig heb geïnformeerd over het genoemde onderzoek.

Als er tijdens het onderzoek informatie bekend wordt die de toestemming van de proefpersoon zou kunnen beïnvloeden, dan breng ik hem/haar daarvan tijdig op de hoogte.

Naam onderzoeker (of diens vertegenwoordiger):

Handtekening: Datum: __ / __ / __

-----------------------------------------------------------------------------------------------------------------

Aanvullende informatie is gegeven door (indien van toepassing):

Naam:

Functie:

Handtekening: Datum: __ / __ / __

-----------------------------------------------------------------------------------------------------------------

* Doorhalen wat niet van toepassing is.
