# Supplementary material for: Physical therapy aimed at self-management versus usual care physical therapy after hip arthroscopy for femoroacetabular impingement: study protocol for a randomized controlled trial
Source: Trials. 2016 Feb 17;17:91. doi: 10.1186/s13063-016-1222-7 (PMC4756499; doi:10.1186/s13063-016-1222-7)
Supplement: Additional file 3: — (A) Specific examples of exercises included in exercise program. (B) Example of exercise progression in exercise program. (DOCX 730 kb) [file 13063_2016_1222_MOESM3_ESM.docx]

**ADDITIONAL FILE 3A Specific examples of exercises included in exercise program.**

| Exercise goal and time of execution | Examples of specific exercises | | | | | |
| --- | --- | --- | --- | --- | --- | --- |
| Self-mobilizations of the hip, pelvis and lumbar spine  Week 0-8 |  |  |  |  |  |  |
| Anterior and posterior hip stretch  Week 2-8 |  | | | | | |
| Hip muscle retraining  Week 0-4 |  |  |  |  |  |  |
| Hip muscle strengthening (focus on extensor/rotator strengthening)  Week 4-14 |  |  |  |  |  |  |
|  |  | |  | | | |
| Functional hip muscle strengthening  Week 10-14 | Exercises based on patient specific goals or (sport) demands such as one leg exercises combined with kicking for soccer or throwing/smashing in combination with one leg stabilization in volleyball/tennis. | | | | | |

**ADDITIONAL FILE 3B Example of exercise progression in exercise program.**

| Example of exercise progression of hip extension | | |
| --- | --- | --- |
|  |  |  |
